# Supplementary material for: Chromatic covalent organic frameworks enabling in-vivo chemical tomography
Source: Nat Commun. 2024 Oct 28;15:9300. doi: 10.1038/s41467-024-53532-7 (PMC11519549; doi:10.1038/s41467-024-53532-7)
Supplement: Supplementary file 1 — Supplementary Information file [file 41467_2024_53532_MOESM1_ESM.pdf]

Supplementary information

Chromatic Covalent Organic Frameworks Enabling In-Vivo Chemical Tomography

Song Wang, [a‡] Yangyang Han, [a‡] Vaishnavi Amarr Reddy, [b] Mervin Chun-Yi Ang, [a] Gabriel Sánchez-Velázquez, [c] Jolly Madathiparambil Saju, [b] Yunteng Cao, [d] Duc Thinh Khong, [a] Praveen Kumar Jayapal, [a] Raju Cheerlavancha, [a] Suh In Loh, [a] Gajendra Pratap Singh, [a] Daisuke Urano, [b] Rajani Sarojam, [b] Benedetto Marelli [a,d]* and Michael S. Strano [a,c]*

[a]Disruptive & Sustainable Technologies for Agricultural Precision, Singapore-MIT Alliance for Research and Technology Centre, Singapore 138602, Singapore

[b] Temasek Life Sciences Laboratory Limited, Singapore 117604, Singapore

[c]Department of Civil and Environmental Engineering, Massachusetts Institute of Technology, Cambridge, MA, 02139, USA

[d]Department of Chemical Engineering, Massachusetts Institute of Technology, Cambridge, MA, 02139, USA

‡These authors contributed equally to this work

Methods

Materials

2,4,6-Tris(4-aminophenyl)pyridine (TAPP, 98%), Tris(4-aminophenyl)amine (TAPA, 98%), 2,4,6-Tris(4-formylphenyl)pyridine (TFPP, 98%), 2,5-

Dimethoxyterephthalaldehyde (DMTA, 99%), 1,3,5-tris(4-aminophenyl)benzene (TAPB, 98%), Tris(4-formylphenyl)amine (TFPA, 98%) were purchased from Jilin Chinese Academy of Sciences - Yanshen Technology Co., Ltd.. Trifluoroacetic acid (99%), Mesitylene (98%), dioxane (99.5%), aniline (99.5%), benzaldehyde (99%), and ethanol (99%) were purchased from Sigma Aldrich Company.

Tobacco (*N. benthamiana*) seeds were obtained from Temasek Life Sciences Laboratory Limited. Tomato (*Lycopersicon esculentum*) seeds were purchase from The Seeds Master company.

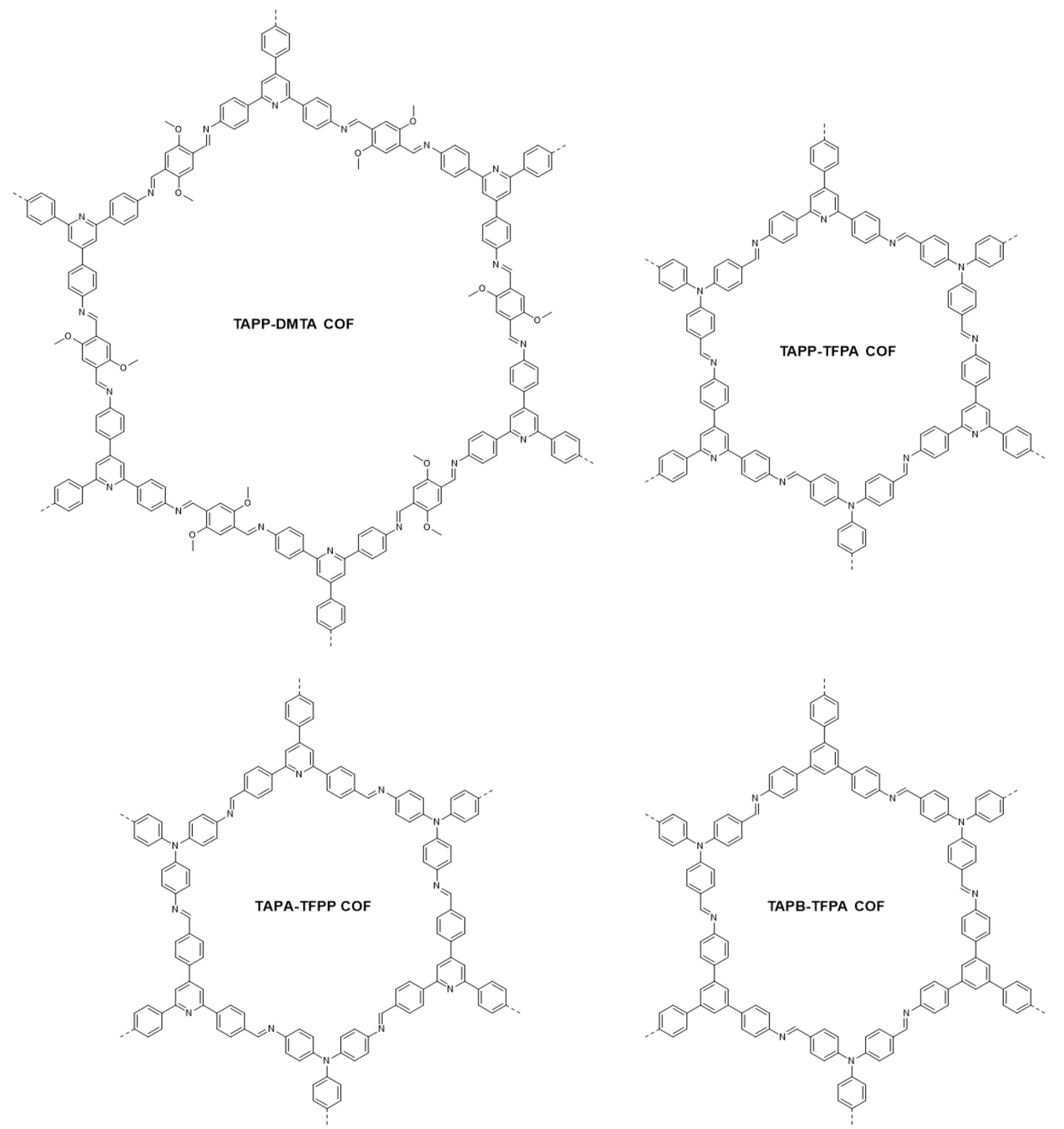

Figure S1 Chemical structures of TAPP-DMTA, TAPP-TFPA, TAPA-TFPP and TAPB-TFPA COFs.

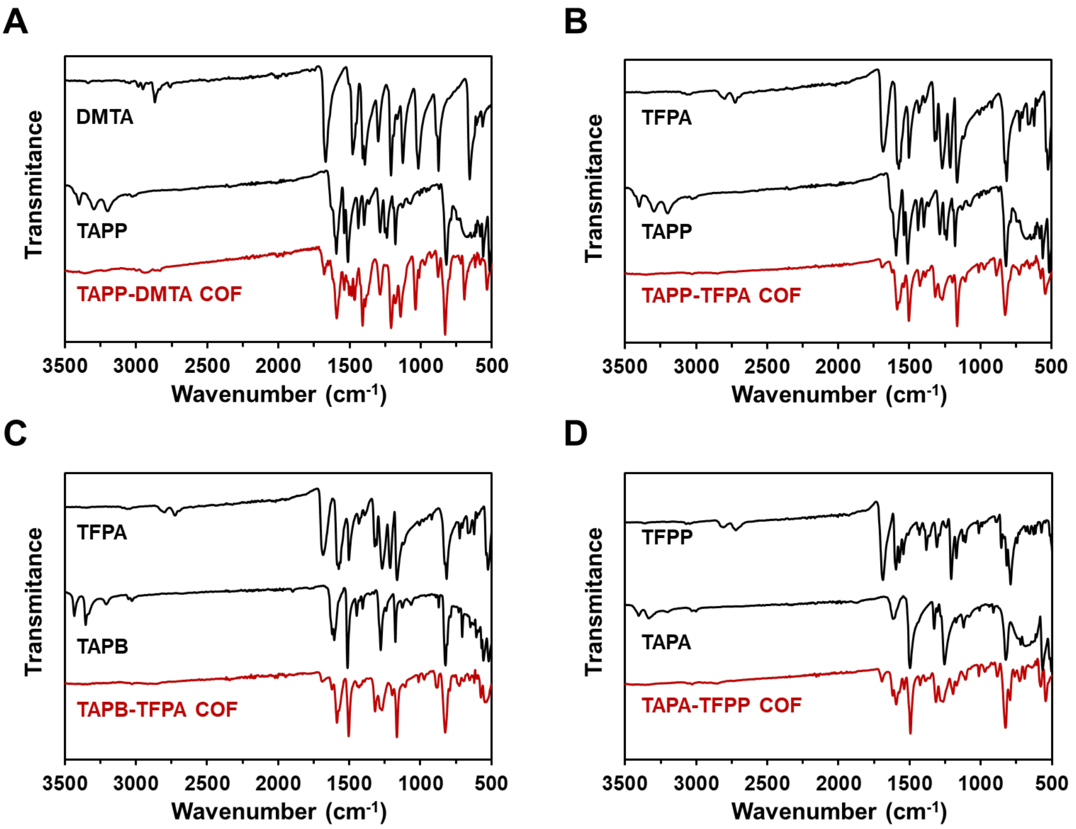

Figure S2 FTIR spectrums of TAPP-DMTA (A), TAPP-TFPA (B), TAPB-TFPA (C) TAPA-TFPP COFs and their corresponding monomers. COFs polymerization through formation of an imine linkage was confirmed by attenuated total reflectance-Fourier transform infrared spectroscopy (ATR-FTIR, Figure S2). When the FTIR spectra of COF powder samples are compared to the ones of monomers, peaks at 3210-3430 cm^{-1} attributed to amine group and peaks at 2710-2830 cm^{-1} attributed to aldehyde group[1] are present in the monomers but disappear in the COFs, while new peaks around 1610 cm^{-1} attributed to imine bond arise, indicating successful polymerization.

COFs crystallinity is an important feature that allows for rapid guest molecule transfer and hence enables fast colorimetric response, when compared with amorphous analogues.[2,3] Their crystallinity was investigated through powder X-ray diffraction analysis (XRD) and predicted stacking models (Figure S2-S5). TAPP-DMTA presented a sharp diffraction peak centered at 2.8° (100) and high ordered diffraction peaks at 5.1° (110), 5.9° (200), 7.2° (210) with lower intensity, which match well with the predicted eclipsed stacking model shown Figure S2. Diffractograms of the other three COFs also depict sharp (100) peaks and clear high ordered diffraction peaks, indicating inclined stacking for both TAPP-TFPA (Figure S3) and TAPB-TFPA (Figure S4), and an eclipsed stacking for TAPA-TFPP (Figure S5).

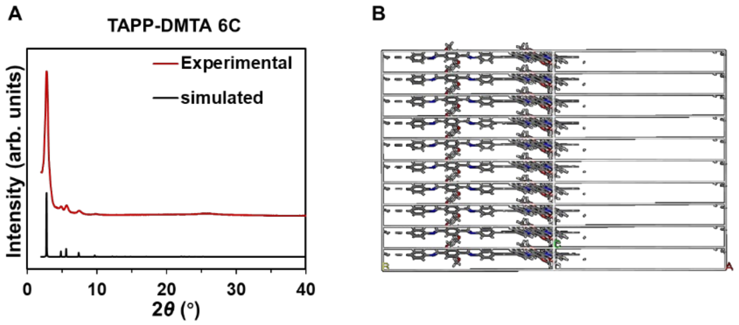

Figure S3 Powder XRD spectrum (A) and simulated crystalline structure (B) of TAPP-DMTA 6C. An eclipsed stacking model is confirmed accordingly. FWHM = 0.55°.

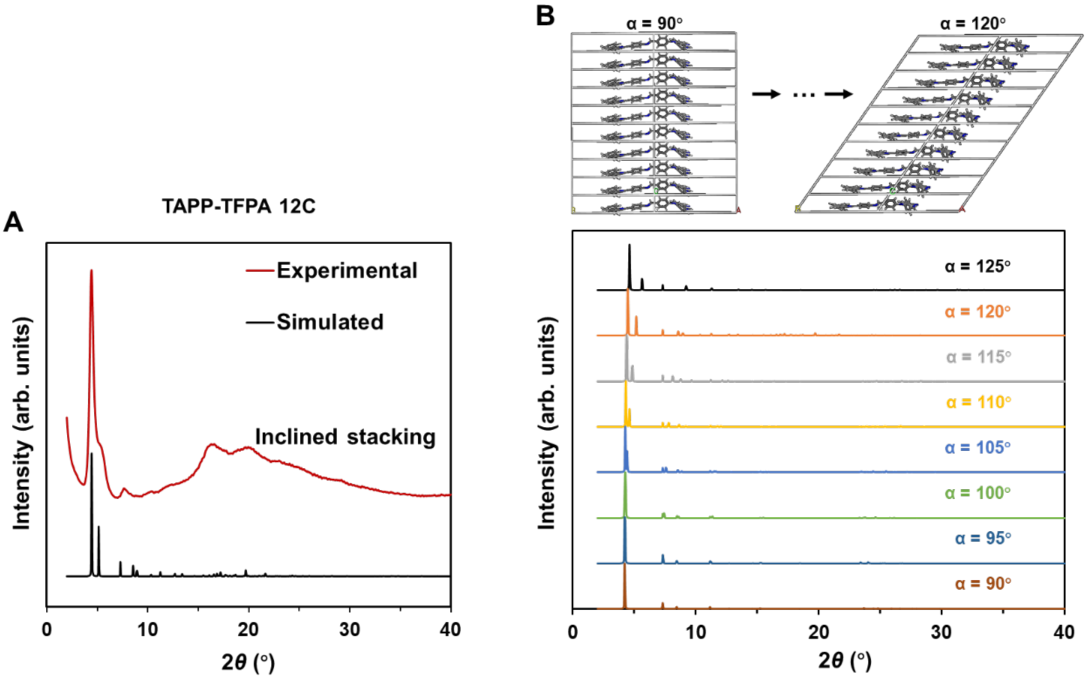

Figure S4 Powder XRD spectrum (A) and simulated crystalline structure (B) of TAPP-TFPA 12C. An inclined stacking model is confirmed accordingly. FWHM = 0.48°.

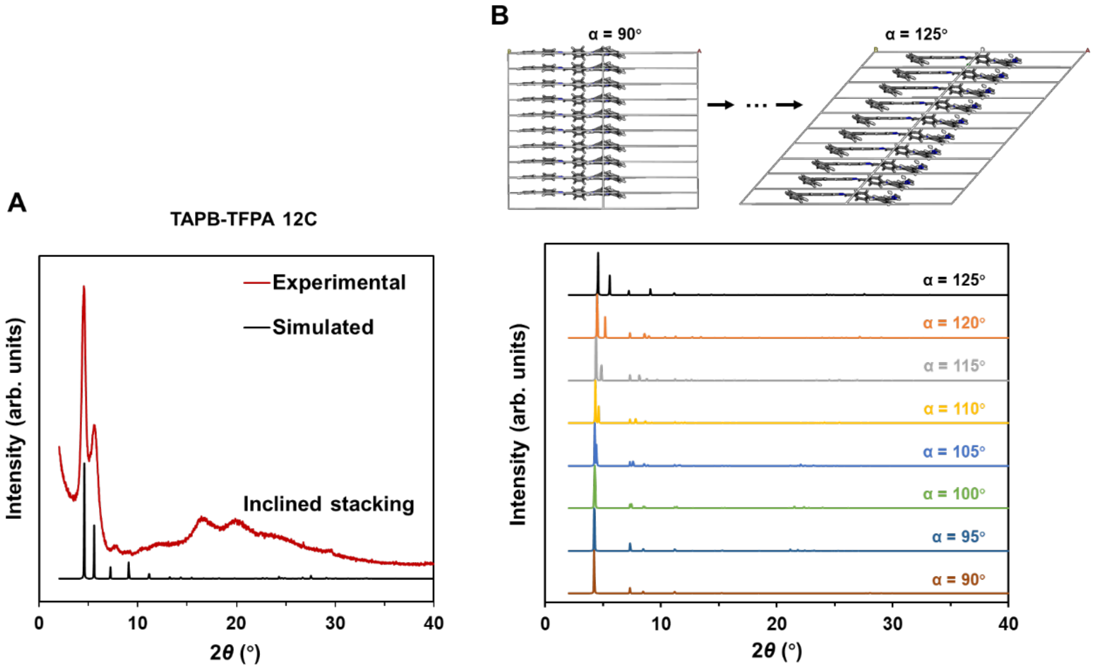

Figure S5 Powder XRD spectrum (A) and simulated crystalline structure (B) of TAPB-TFPA 12C. An inclined stacking model is confirmed accordingly. FWHM = 0.47°.

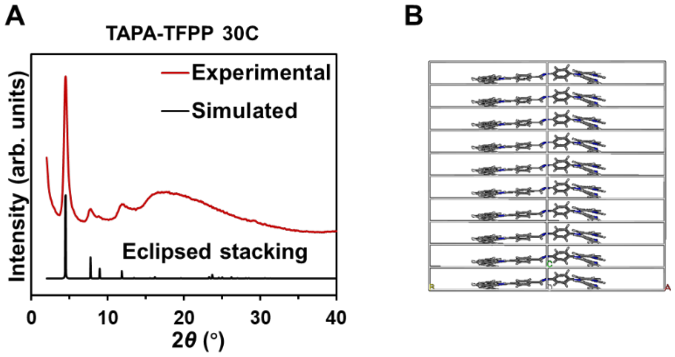

Figure S6 Powder XRD spectrum (A) and simulated crystalline structure (B) of TAPA-TFPP 30C. An eclipsed stacking model is confirmed accordingly. FWHM = 0.55°.

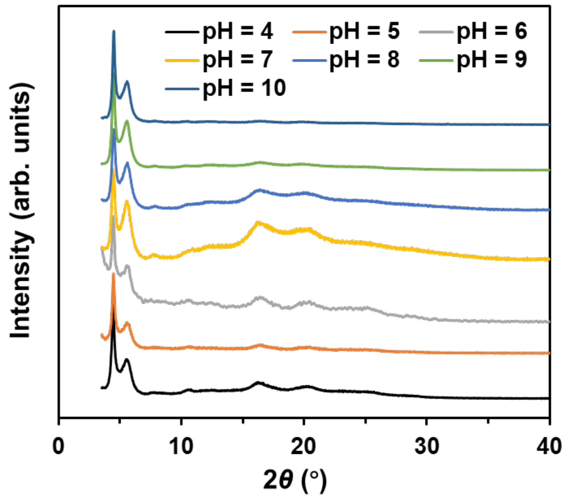

Figure S7 XRD spectrum of TAPP-TFPA powders after soaking in aqueous buffer solutions with pH values from 4 to 10 for 1 day.

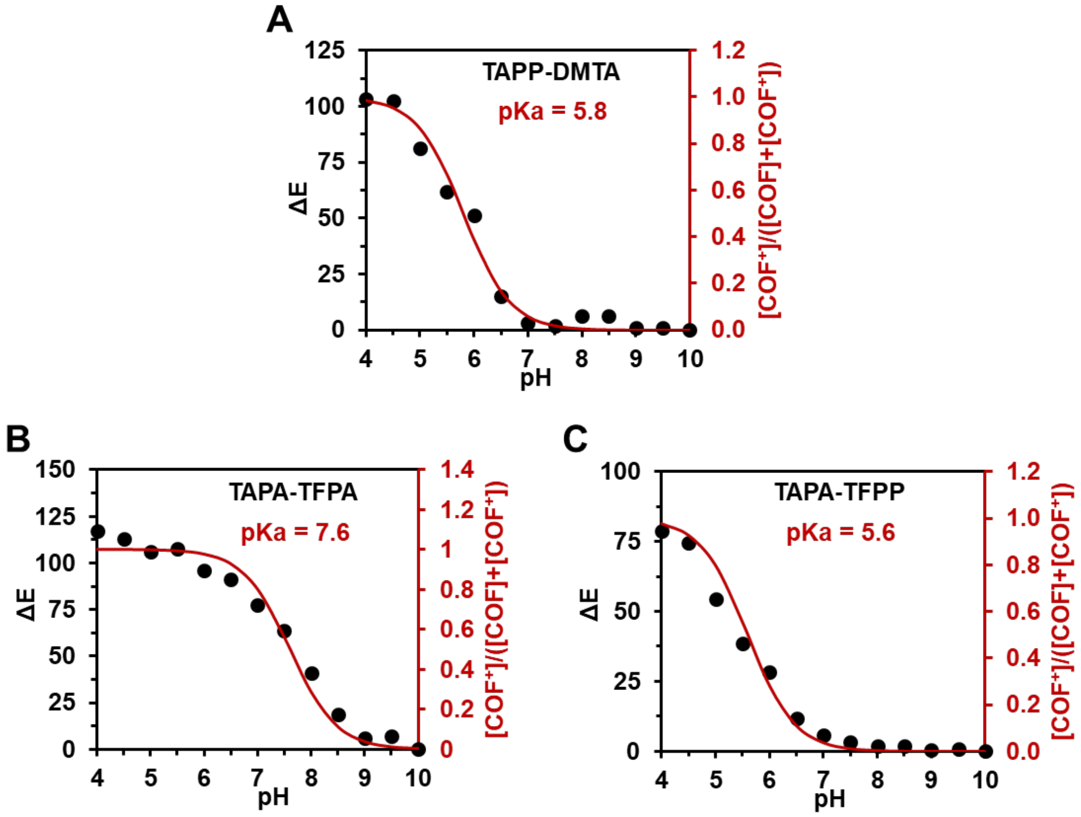

Figure S8 ΔE varied with pH value for TAPP-DMTA (A), TAPA-TFPA (B) TAPA-TFPP (C) and fitted pKa curve according to Henderson-Hasselbalch equation.

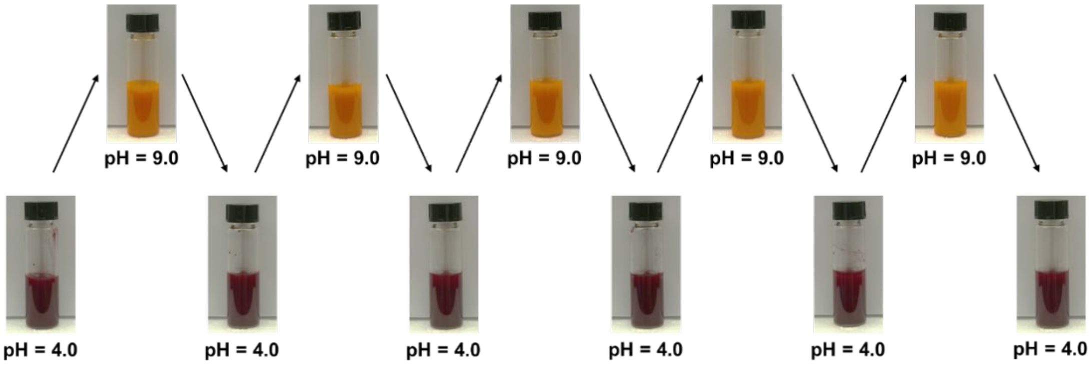

Figure S9 Photos of TAPP-TFPA COF powder aqueous dispersion during 10 times buffer switching.

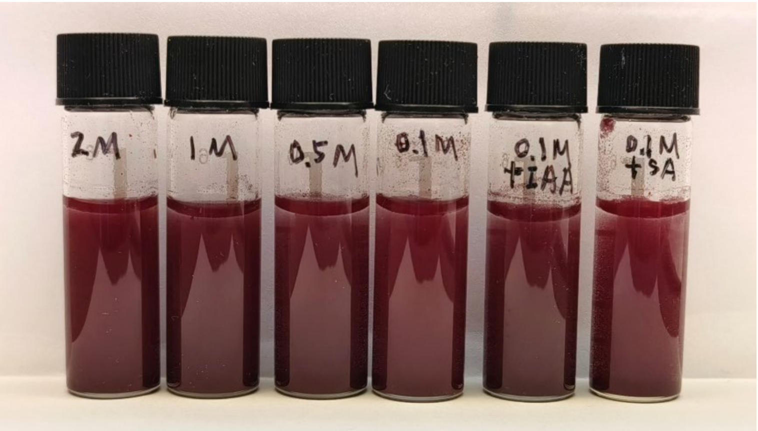

Figure S10 Photos of TAPP-TFPA COF powders dispersed in sodium acetate buffer solutions (pH=5.2) with different concentrations from 0.1 M to 2 M, as well as the presence of indole-3-acetic acid (IAA) and salicylic acid (SA) with a concentration of 1 mg/mL.

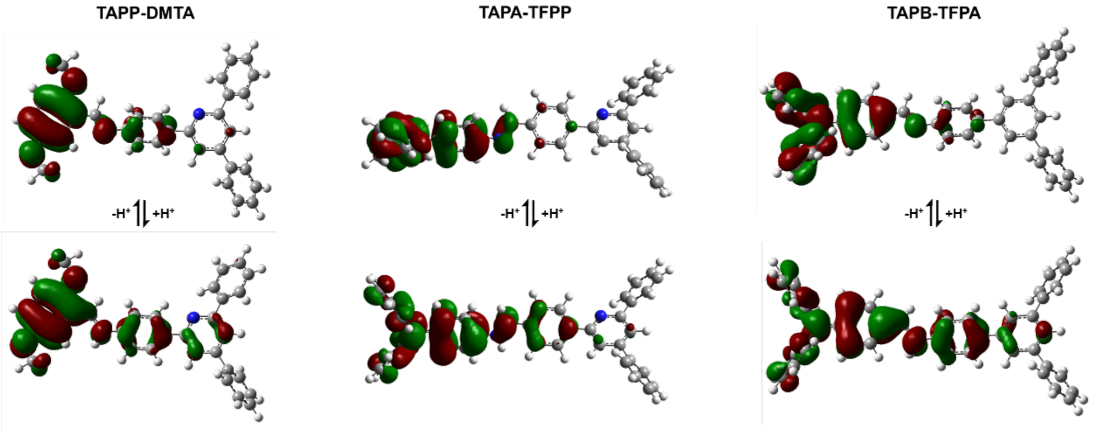

Figure S11 Density functional theory (DFT) optimized molecular structures and highest occupied molecular orbitals (HOMOs) of unprotonated and protonated TAPP-DMTA, TAPA-TFPP and TAPB-TFPA repeat units.

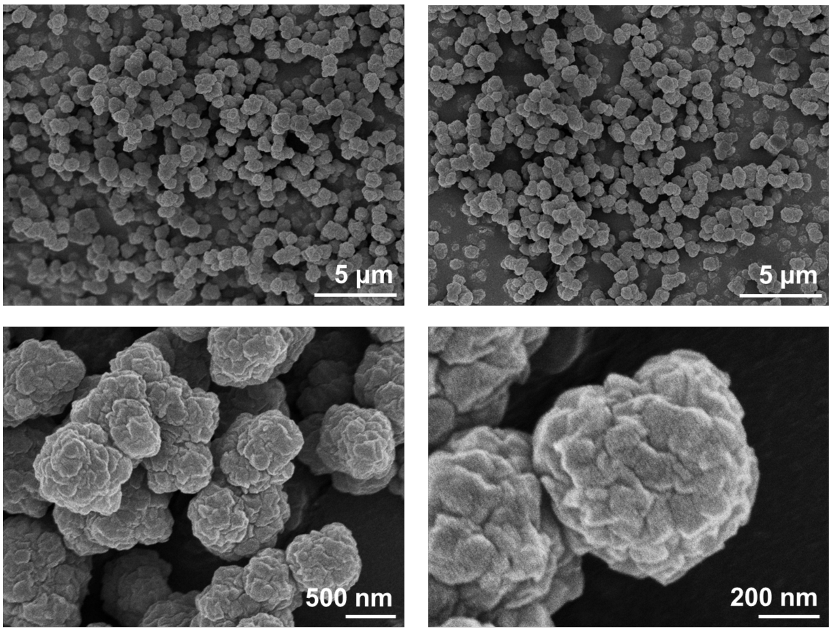

Figure S12 SEM images of TAPP-TFPA powders synthesized with 3 equiv. competitors.

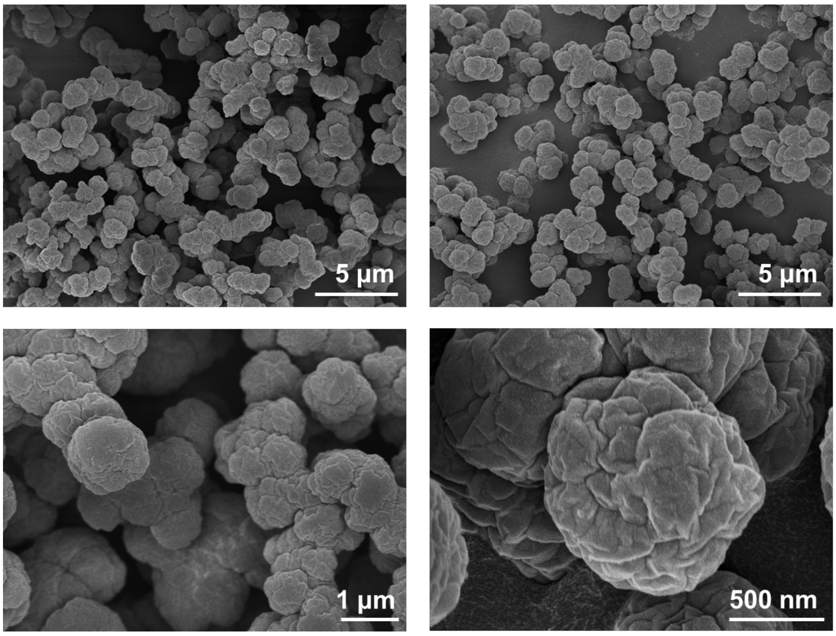

Figure S13 SEM images of TAPP-TFPA powders synthesized with 6 equiv. competitors.

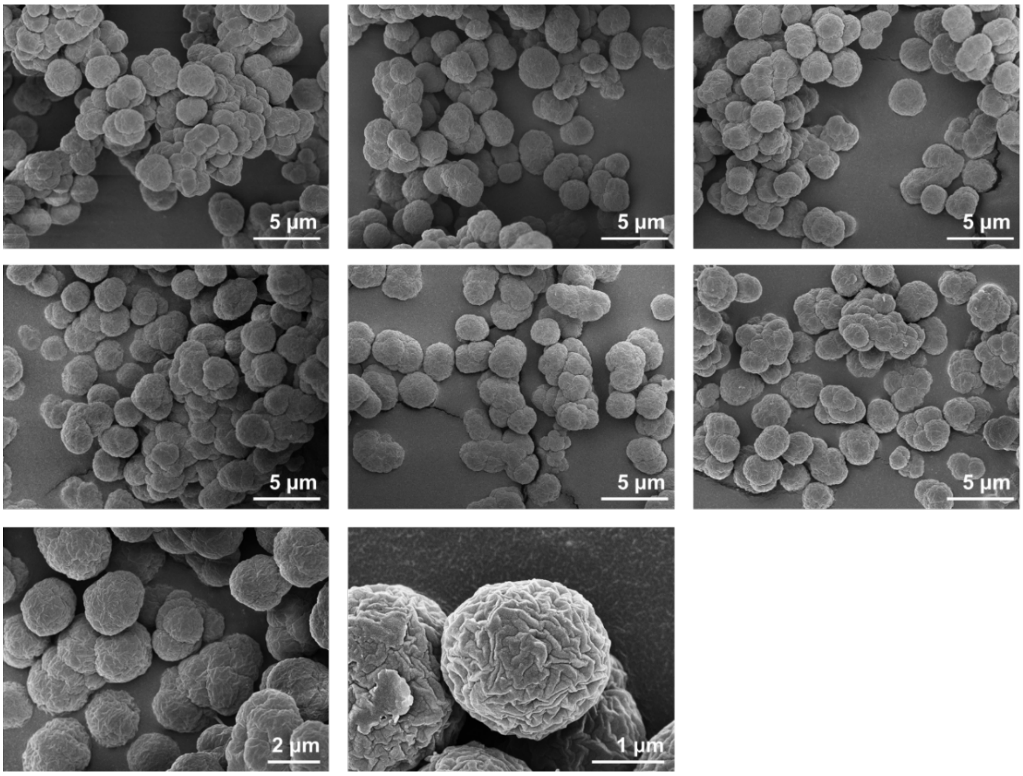

Figure S14 SEM images of TAPP-TFPA powders synthesized with 9 equiv. competitors.

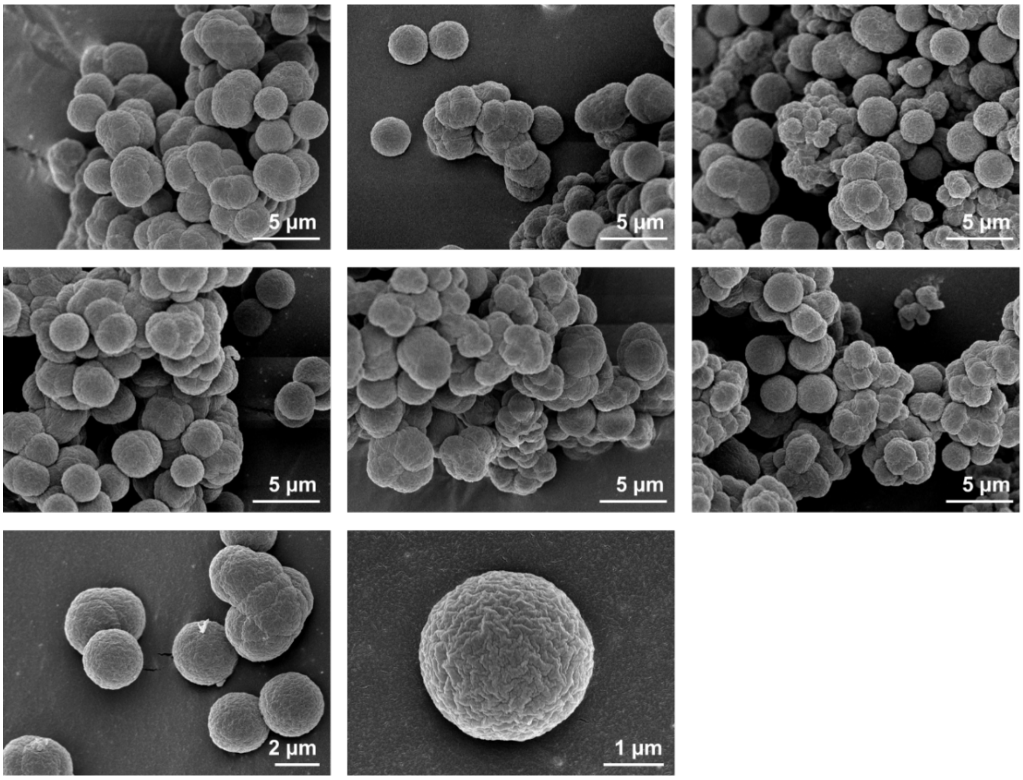

Figure S15 SEM images of TAPP-TFPA powders synthesized with 12 equiv. competitors.

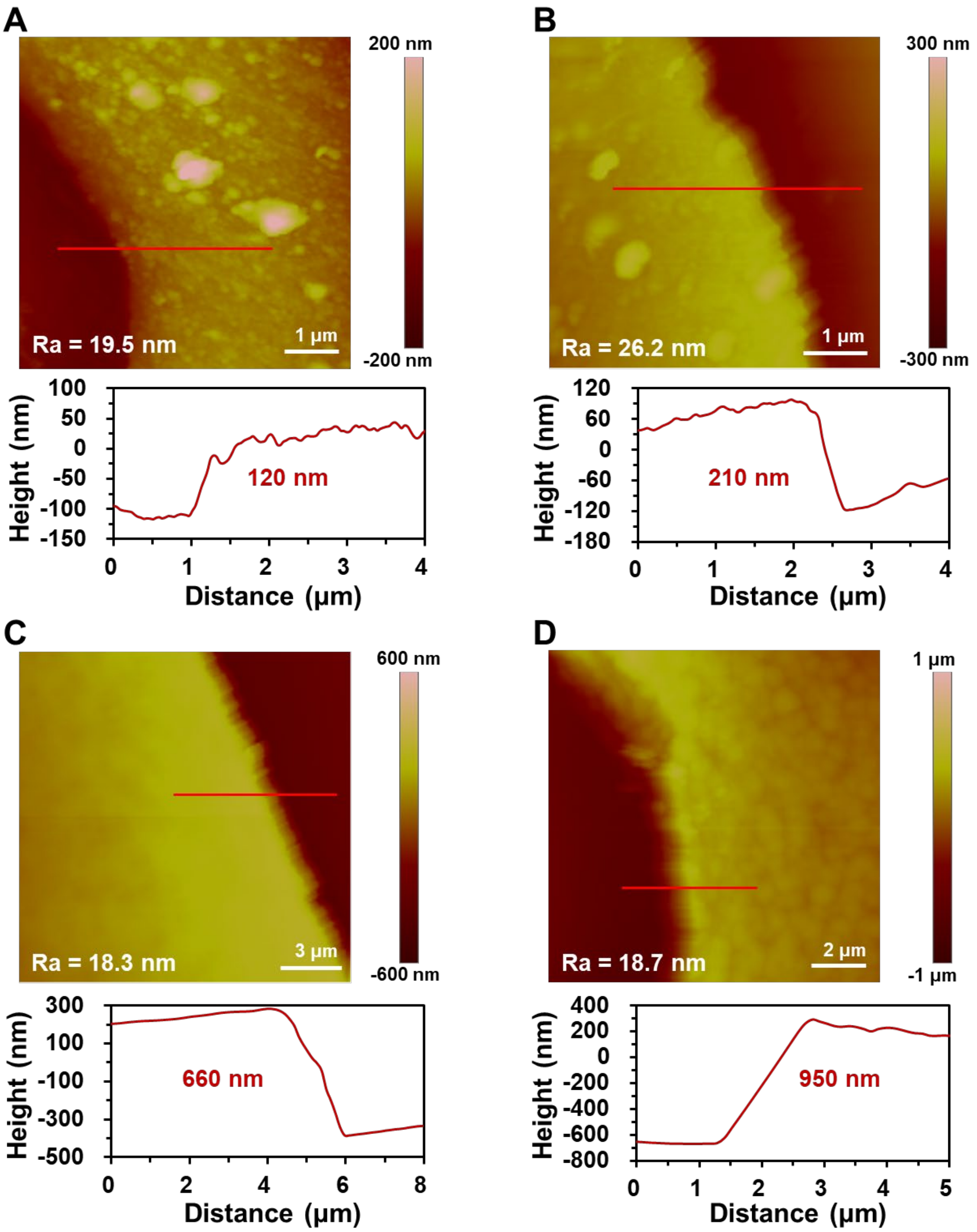

Figure S16 AFM images and heigh profiles of TAPP-TFPA film growing on PET substrates synthesized with 3 (A), 6 (B), 9 (C) and 12 (D) equiv. competitors.

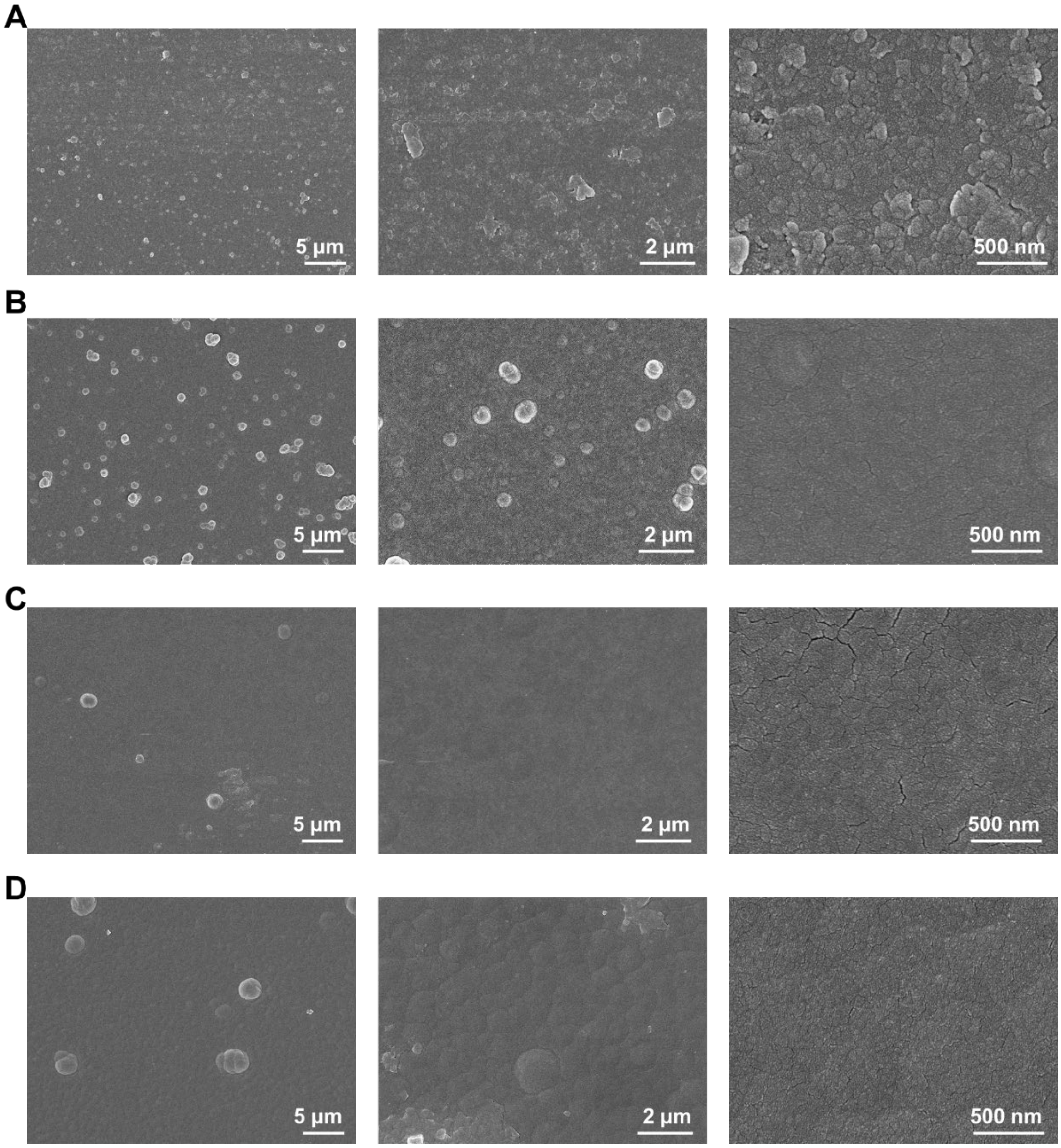

Figure S17 SEM images of TAPP-TFPA film growing on PET substrates synthesized with 3 (A), 6 (B), 9 (C) and 12 (D) equiv. competitors.

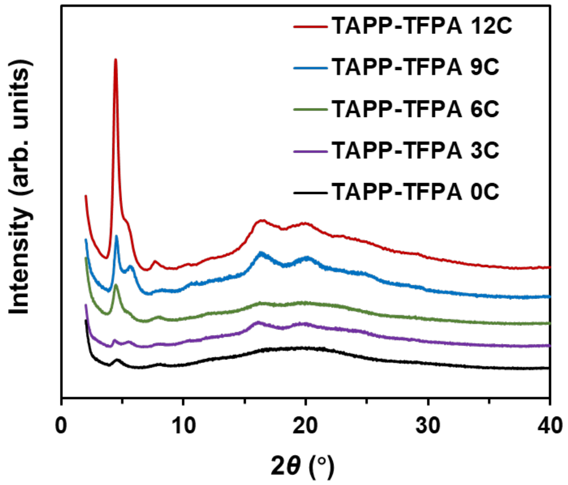

Figure S18 XRD spectrum of TAPP-TFPA synthesized with different competitor amounts (C, equivalent over TAPP monomer) from 0C to 12C.

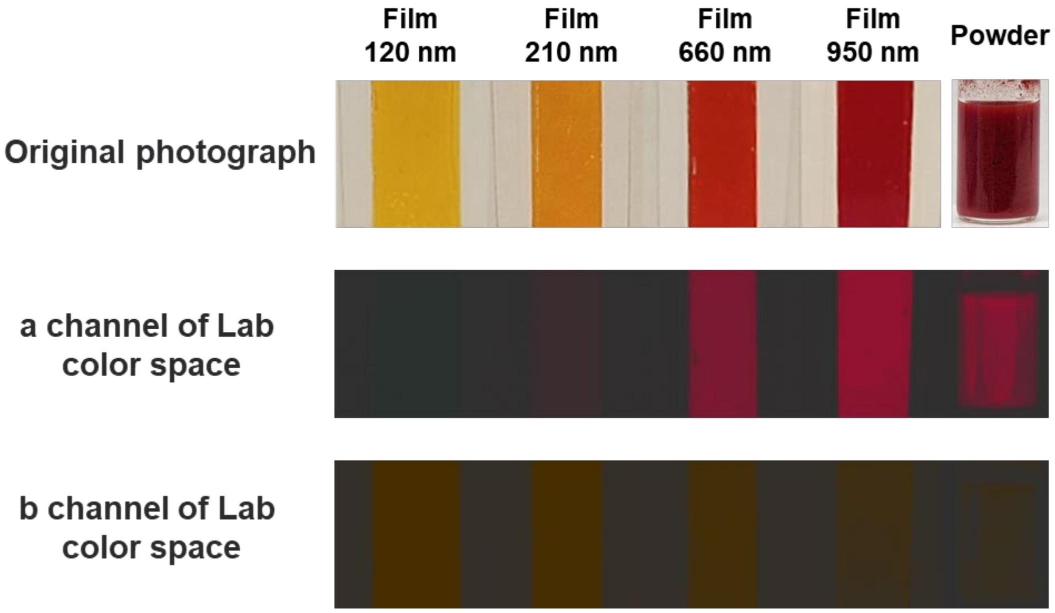

Figure S19 Original photograph, a and b channel of Lab color space for TAPP-TFPA powder and films with different thickness in a buffer with pH = 5.0.

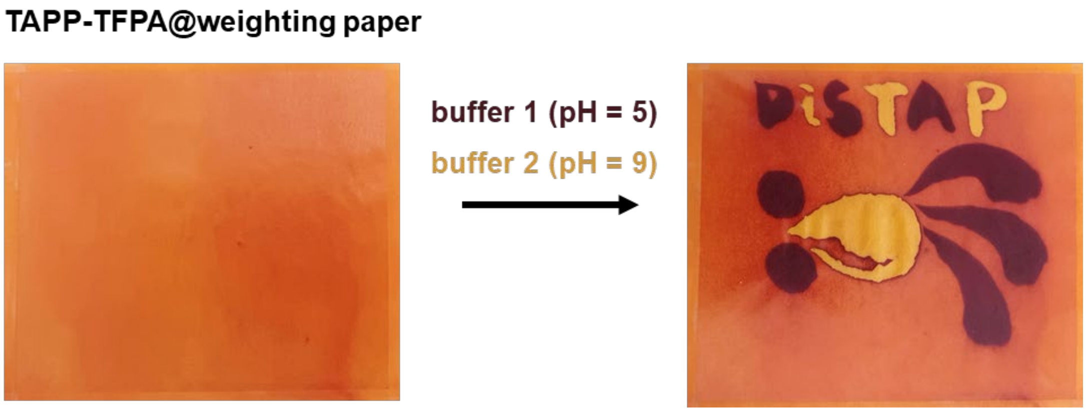

Figure S20 (A) TAPP-TFPA@weighting paper present obvious color difference in responding to acid and base buffers.

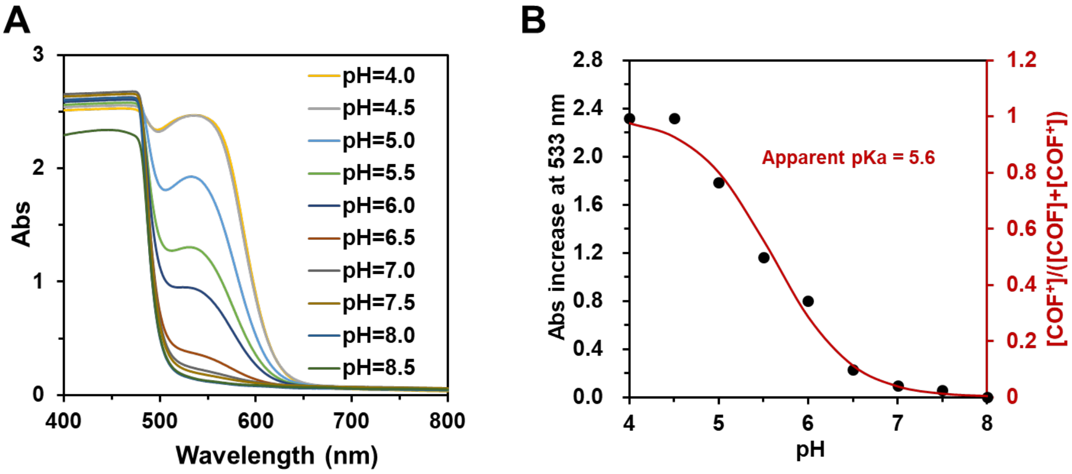

Figure S21 (A) UV-Vis absorption spectrums of TAPP-TFPA film with a thickness of 950 nm on PET film in response of different pH ranging from 4.0 to 8.5. (B) Absorption increase at 533 nm varying with pH value and fitting with Henderson-Hasselbalch equation. Apparent pKa of 5.6 was obtained for TAPP-TFPA film with thickness of 950 nm.

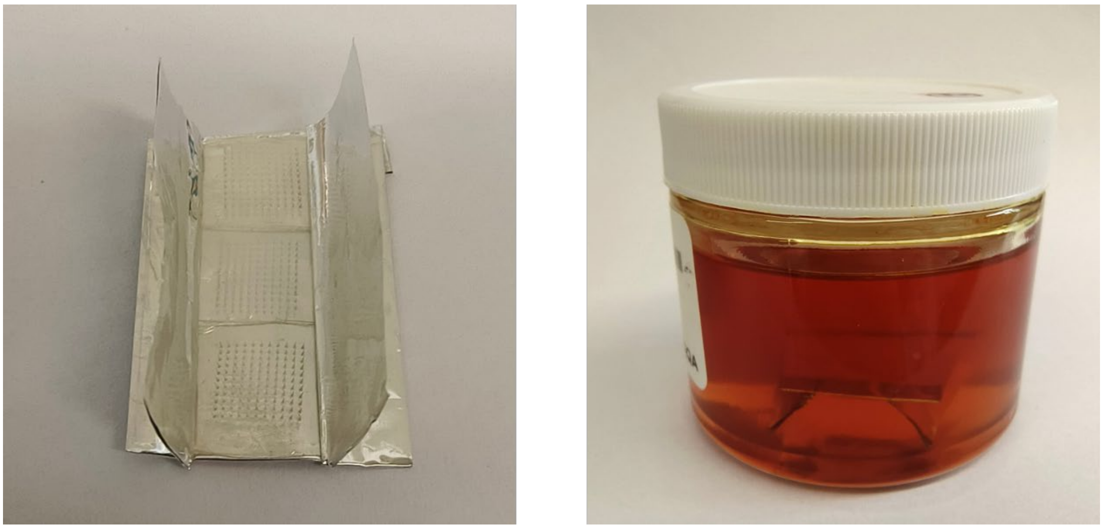

Figure S22 Photos to illustrate TAPP-TFPA coating on silk microneedles. Silk microneedle array was held by an aluminum foil and placed upside down in TAPP-TFPA synthetic solution.

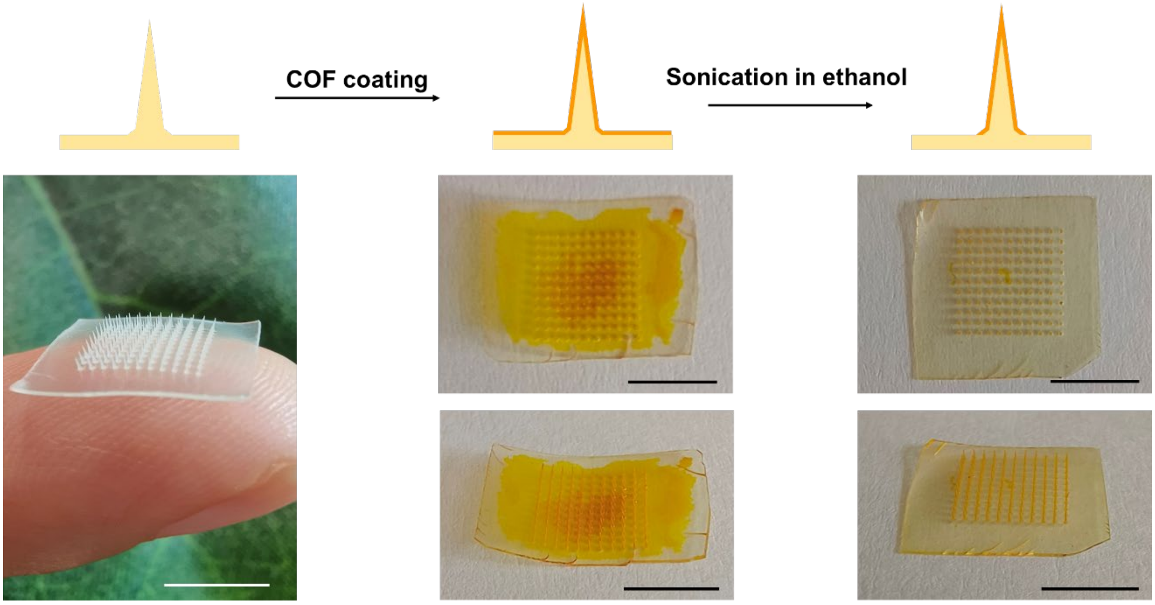

Figure S23 Illustration and photos of silk microneedle array and TAPP-TFPA silk microneedle array before and after selectively removing TAPP-TFPA on substrate. We attribute the selectively removal of the COF layer to the morphological difference between the rough tip body and the flat tip substrates, that results in lower Van der Waals affinity between SF and the COF layer in the body due to smaller interface area. Scale bar: 5 mm.

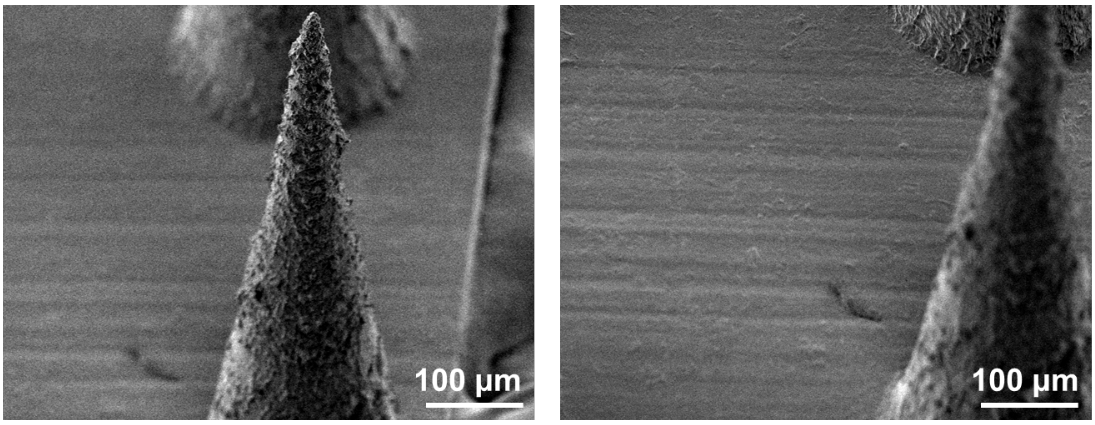

Figure S24 SEM images of silk microneedle. The surface of microneedle is much rougher than that of substrate.

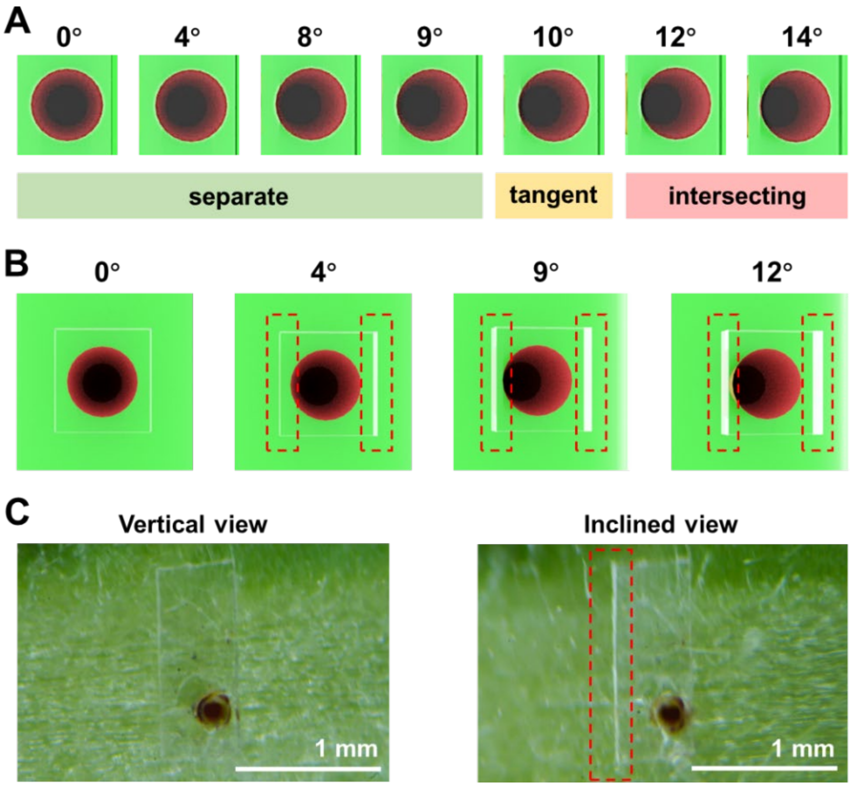

Figure S25 (A) Simulated top views of the microneedle with different viewing angle deviation. At viewing angle deviation of ~10°, the circles tangent, which means one side of the information is missing due to the overlapping. So, for the microneedle we used, the viewing angle deviation need to be kept within ~9°. (B) Simulated top views of the microneedle substrate edge with different viewing angle deviation edge. From the perfect vertical view, the edge of the microneedle substrate can not be observed. With the viewing angle deviation increase, more and more edge can be seen. (C) Experimental microscope photo of TSMN700 injecting into tobacco leave. In experiment, we try our best to get the top view photos with minimum substrate edge showing up.

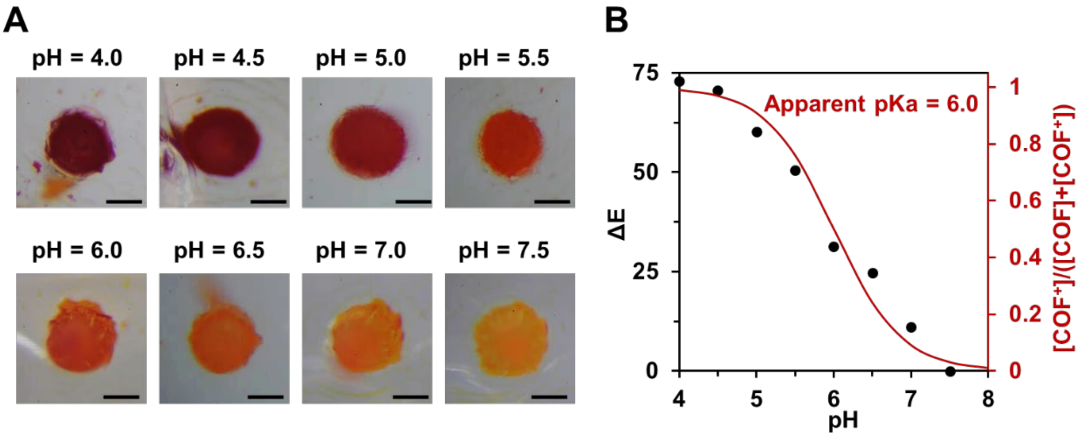

Figure S26 Top view optical microscope images (A) and ΔE (B) of TSMN700 upon exposure to buffers with pH values varying from 4.0 to 7.5. ΔE is calculated based on TSMN700 color at pH = 7.5 buffer. Scale bar: 100 μm.

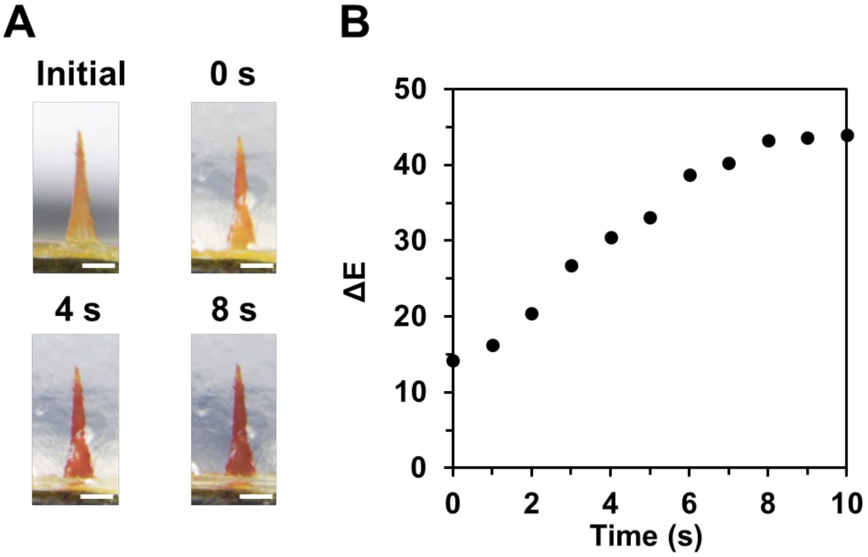

Figure S27 Side views optical microscope images (A) and ΔE (B) of TSMN700 as a function of time upon insertion into a transparent acidic gel (pH 5). TSMN700 is directly drying from ethanol without any treatment. ΔE is calculated based on initial TSMN700. Scale bar: 200 μm.

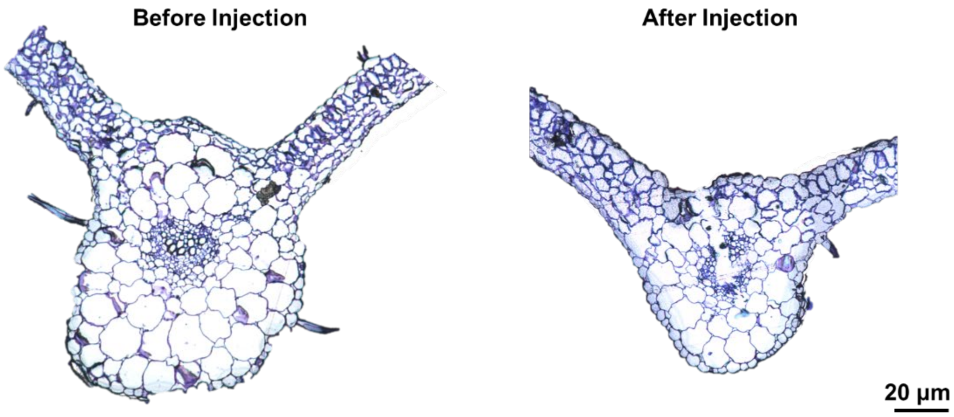

Figure S28 Tobacco leaf midrib cross sections stained with toluidine blue showing before (top) and after (down) injection at abaxial side. Blue circle indicates vasculature zone.

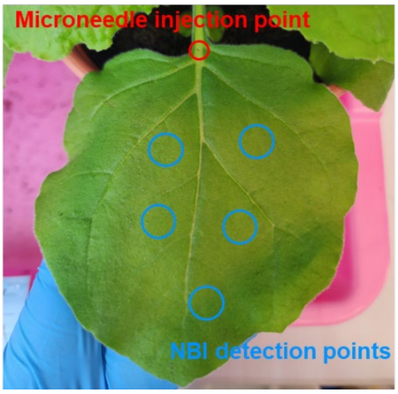

Figure S29 Microneedle injection point and NBI detection points for the study of healthy influence of microneedle on tobacco.

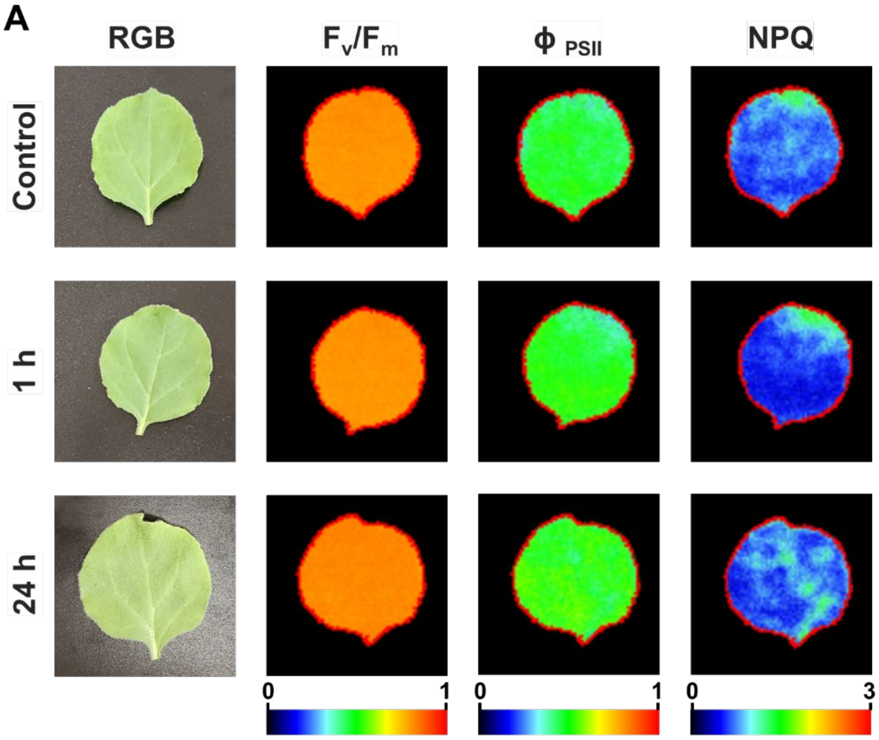

B

Runs	F_v/F_m			Φ PSII			NPQ		
	Control	1 h	24 h	Control	1 h	24 h	Control	1 h	24 h
1	0.82	0.82	0.82	0.39	0.49	0.49	1.36	0.90	0.80
2	0.81	0.81	0.82	0.46	0.48	0.51	0.77	0.67	0.76
3	0.82	0.81	0.83	0.55	0.47	0.53	0.63	0.90	0.74
avg.	0.82	0.81	0.82	0.47	0.48	0.51	0.92	0.82	0.77

Figure 30 (A) Representative images of the digital and symptomatology photos of control leaf (without injection) and experimental leaves after injection of microneedle after 1 h and 24 h. (B) PSII efficiency (F_V/F_M and Φ_{PSII}) and non-photochemical quenching (NPQ) values are summarized in the table.

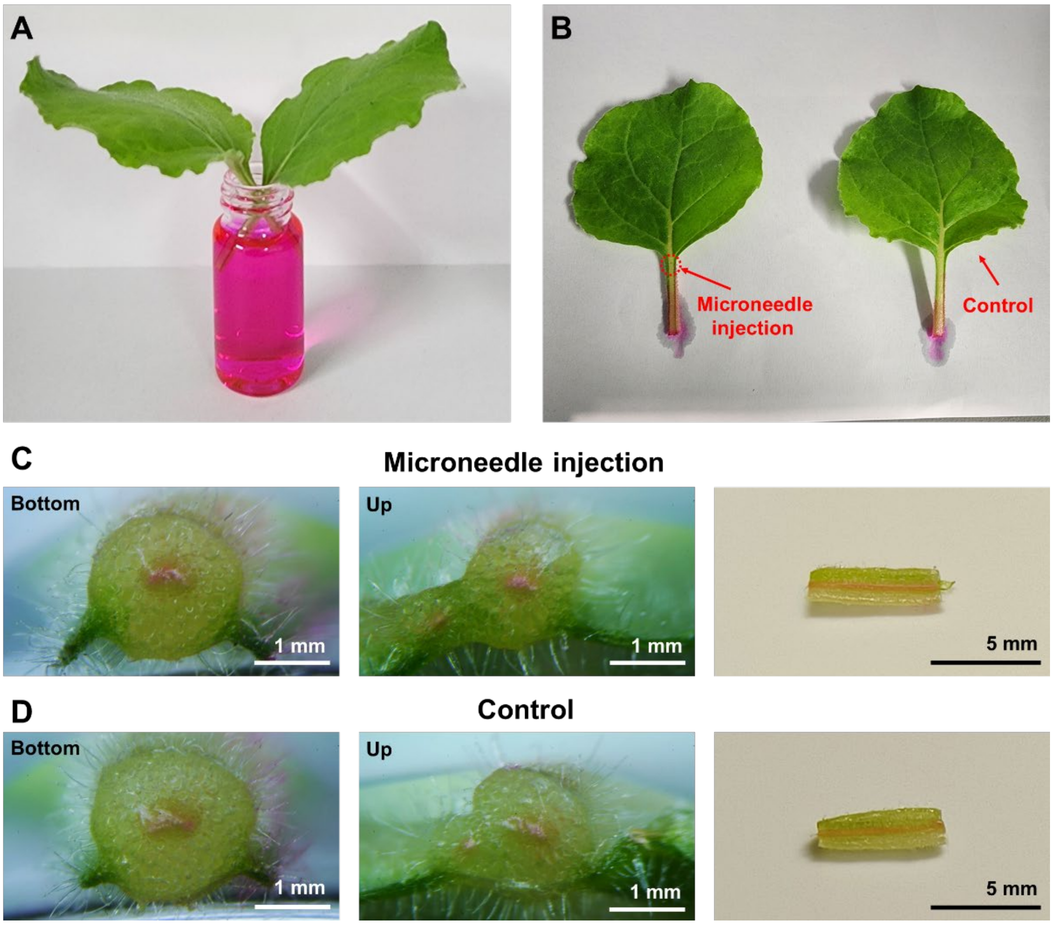

Figure S31. Digital photos showing the rhodamine B transporting in tobacco leaves. Tobacco leaf with and without microneedle injection was cut off and its petiole was submerged into rhodamine B (100 μM) solution for 5 minutes (A). The rhodamine B solution was absorbed by the tobacco leaves (B). The petiole of leaves with or without microneedle injection was cut at 5 mm above and below the injection site and the cross-section was observed by microscope (left and middle figures in C and D). Rhodamine B transported and remained in the vascular bundles of the midrib at 5-10 mm above the injection point (right photos in C and D). Vascular bundles of the midrib were exposed by cutting. The vascular bundles of the petiole, whether injected with microneedle (C) or not (D), were stained with rhodamine B.

Supplementary References

1 Luzgin, M. V., Thomas, K., van Gestel, J., Gilson, J.-P. & Stepanov, A. G. Propane carbonylation on sulfated zirconia catalyst as studied by 13C MAS NMR and FTIR spectroscopy. *Journal of Catalysis* **223**, 290-295 (2004).

2 Liu, X. *et al.* Recent advances in covalent organic frameworks (COFs) as a smart sensing material. *Chemical Society Reviews* **48**, 5266-5302 (2019).

3 She, P., Qin, Y., Wang, X. & Zhang, Q. Recent progress in external-stimulus-responsive 2D covalent organic frameworks. *Advanced Materials* **34**, 2101175 (2022).
