# Supplementary material for: Chromatic covalent organic frameworks enabling in-vivo chemical tomography
Source: Nat Commun. 2024 Oct 28;15:9300. doi: 10.1038/s41467-024-53532-7 (PMC11519549; doi:10.1038/s41467-024-53532-7)
Supplement: Supplementary file 4 — Description of Additional Supplementary Files [file 41467_2024_53532_MOESM4_ESM.pdf]

Supplementary Movie 1:
The microscopic side views of TSMN700 as a function of time upon insertion into a transparent acidic gel (pH 5).
